# Population phylogenomic analysis of mitochondrial DNA in wild boars and domestic pigs revealed multiple domestication events in East Asia

**DOI:** 10.1186/gb-2007-8-11-r245

**Published:** 2007-11-19

**Authors:** Gui-Sheng Wu, Yong-Gang Yao, Kai-Xing Qu, Zhao-Li Ding, Hui Li, Malliya G Palanichamy, Zi-Yuan Duan, Ning Li, Yao-Sheng Chen, Ya-Ping Zhang

**Affiliations:** 1 State Key Laboratory of Genetic Resources and Evolution, Kunming Institute of Zoology, Chinese Academy of Sciences, Kunming 650223, China; 2 Laboratory for Conservation and Utilization of Bio-resource, Yunnan University, 2 North Greenlake Street, Kunming 650091, China; 3 The Graduate School of the Chinese Academy of Sciences, 19 Yuquan Street, Beijing, 100039, China; 4 Center for Pharmacogenomics, Department of Psychiatry and Behavioral Science, University of Miami Miller School of Medicine, 1580 NW 10^th ^Ave., Miami, Florida 33136, USA; 5 Key Laboratory of Animal Models and Human Disease Mechanisms, 32 East Jiaochang Road, Kunming Institute of Zoology, Chinese Academy of Sciences, Kunming 650223, China; 6 China Agriculture University, 2 West Yuanmingyuan Street, Beijing 10094, China; 7 College of Life Sciences, Sun Yat-sen University, 135 West Xin'gang Street, Guangzhou 510275, China

## Abstract

**Background:**

Previously reported evidence indicates that pigs were independently domesticated in multiple places throughout the world. However, a detailed picture of the origin and dispersal of domestic pigs in East Asia has not yet been reported.

**Results:**

Population phylogenomic analysis was conducted in domestic pigs and wild boars by screening the haplogroup-specific mutation motifs inferred from a phylogenetic tree of pig complete mitochondrial DNA (mtDNA) sequences. All domestic pigs are clustered into single clade D (which contains subclades D1, D2, D3, and D4), with wild boars from East Asia being interspersed. Three haplogroups within D1 are dominant in the Mekong region (D1a2 and D1b) and the middle and downstream regions of the Yangtze River (D1a1a), and may represent independent founders of domestic pigs. None of the domestic pig samples from North East Asia, the Yellow River region, and the upstream region of the Yangtze River share the same haplogroup status with the local wild boars. The limited regional distributions of haplogroups D1 (including its subhaplogroups), D2, D3, and D4 in domestic pigs suggest at least two different *in situ *domestication events.

**Conclusion:**

The use of fine-grained mtDNA phylogenomic analysis of wild boars and domestic pigs is a powerful tool with which to discern the origin of domestic pigs. Our findings show that pig domestication in East Asia mainly occurred in the Mekong region and the middle and downstream regions of the Yangtze River.

## Abstract 2

A fine-grained mitochondrial DNA phylogenomic analysis was conducted in domestic pigs and wild boars, revealing that pig domestication in East Asia occurred in the Mekong and the middle and downstream regions of the Yangtze river.

## Background

The origin and dispersal of major domestic animals have been widely studied in recent years and great progress has been made [1-18]. Multiple origin has been revealed to be a common phenomenon in domestic animals such as cattle, goats, chicken, and horses [7-9,12,17,19]. Several studies have shown that pigs were independently domesticated in various parts of the world [5,16,20-25]. The time of divergence between European and Asian pig mitochondrial DNAs (mtDNAs) was long before the time of possible pig domestication, which supported the independent origin of domestic pigs in Europe and Asia [5,26]. By analyzing the mtDNA control region (D-loop) sequences of worldwide wild boars, domestic pigs, and ancient specimens, recent studies conducted by Larson and coworkers [16,27,28] have revealed a schematic profile concerning the origin of wild boars and their dispersal and domestication across Eurasia, as well as the Neolithic expansion in Island South East Asia and Oceania. However, because of the small sample size from East Asia and the limited resolution of phylogeny based on partial mtDNA D-loop sequences of pigs, a detailed picture of the origin and dispersal of domestic pigs in East Asia is still to be developed.

To investigate where the East Asian pigs were domesticated and to reconstruct their early dispersal history, we conducted a population phylogenomic analysis of wild boars and domestic pigs by applying a strategy consisting of the following steps. First, we sequenced 670 base pair (bp) fragments of the mtDNA D-loop region in 567 domestic pigs and 155 wild boars across China, South East Asia, and India. Then, we selected 24 wild boars and domestic pigs and analyzed their entire mtDNA sequences. Each of these 24 samples represented a unique haplotype in the major clades observed in a neighbor-joining (NJ) tree of the 722 D-loop sequences (Additional data file 1). Employing a strategy that has been well described by us and others in anthropologic studies [29-38], haplogroup-specific mutation motifs (a string of characteristic mutations shared exclusively by its members) for the respective haplogroups (monophyletic groups or clades in the tree) were inferred from the phylogenetic tree of 42 (near) complete Asian pig mtDNA sequences determined in this study and from published sources. The haplogroup-specific motifs were further screened in all domestic pigs and wild boars to justify the inferred haplogroup status of each sample based on the available information. Finally, all our samples and previously published mtDNA data were assigned to haplogroups based on the haplogroup-specific mutation motifs in the sequence. This fine-grained phylogeographic analysis of matrilineal components of wild boars and domestic pigs provided new insights into the origin and domestication of pigs in East Asia.

## Results and discussion

mtDNA control region sequences (670 bp) of 567 domestic pigs and 155 wild boars across China, South East Asia, and India were determined. A preliminary phylogenetic analysis of the 119 haplotypes of these D-loop sequences was performed and revealed several clades in the tree (Additional data file 1). Then, the complete mtDNAs of 24 samples of wild boars and domestic pigs, each representing a unique haplotype in the major clades in this tree, were selected and sequenced. It should be noted that although the choice of the specific representative samples within certain clade was selected at random, each sample from the same clade has equal potential to allow us to determine the schematic backbone and mutation motif of this clade/haplogroup. The African warthog is well known to be distinct from Eurasian wild boars and has frequently been used as the outgroup in previous phylogenetic studies of pigs [16,25,26,39-42]. In the present study we completely sequenced an mtDNA of African warthog (*Phacochoerus africanus*) and used it as the outgroup to root the mtDNA genome tree.

**Figure 1:**
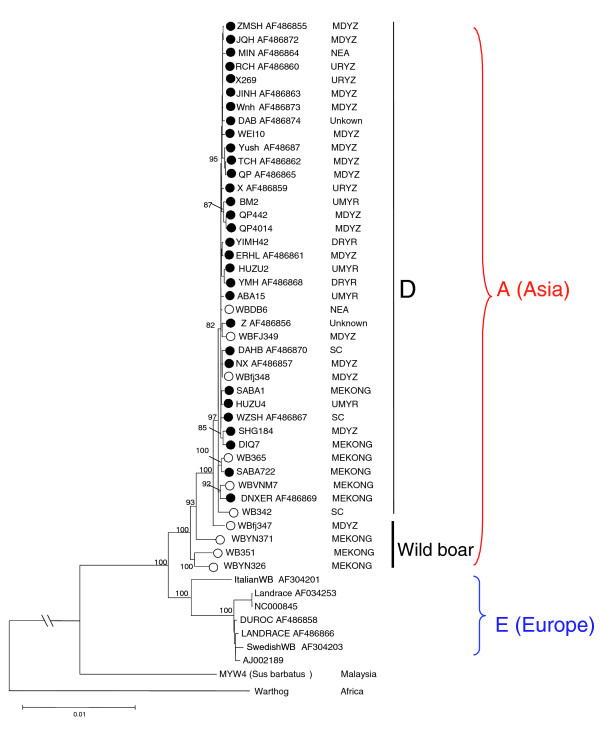
Phylogenetic tree. Shown is a phylogenetic tree of wild boars and domestic pigs from Asia and Europe based on 50 (near) complete mitochondrial DNA (mtDNA) sequences. The tree was constructed by the neighbor-joining method with an Africa warthog as the outgroup. The numbers indicated at the nodes were bootstrap supports based on 1,000 replicates. East Asian domestic pigs and wild boars were marked by filled circles and open circles, respectively.

The NJ tree of 50 mtDNA genomes (including 24 published near complete sequences; Additional data file 2) revealed two major clades, E and A, which represent the wild boars (*Sus scrofa*) and domestic pigs from Europe and East Asia, respectively (Figure 1). Within clade A, all Asian domestic pig mtDNAs were further clustered into a single clade D, with wild boars from this region intermingled (Figure 1 and Additional data file 3). Phylogenetic trees constructed using other methods, such as maximum parsimony and Bayesian estimation, exhibited similar topology for all major clades in the NJ tree (Additional data file 4) and further confirmed the monophyletic position of East Asian domestic pigs and wild boars. The phylogenetic position of the newly sequenced Malaysia wild boar (*Sus barbatus*) fell outside the macro-clade containing Eurasian samples.

The information read from the mtDNA genome tree enabled us to conduct a phylogenomic analysis for wild boars and domestic pigs. By detecting the haplogroup unique mutation motif (Additional data file 3), each mtDNA could be allocated to the smallest named haplogroup to which it belongs. For instance, haplogroup D1 was characterized by five mutations at sites 500, 2374, 11432, 12064, 16301, whereas its subhaplogroup D1a was defined further by the additional variant at site 14198 (Additional data file 3). By screening these haplogroup-specific mutations in each mtDNA, it could reliably be classified into haplogroup D1 based on the presence of the five D1 specific mutations and further into D1a if it also harbored the mutation at site 14198. Based on our established haplogroup classification system, published pig mtDNA cytochrome (Cyt) *b *and D-loop sequences (Additional data file 5) were also tentatively classified by haplogroup-specific motif recognition and/or a matching or near-matching strategy [30,34,35,38] with the mtDNAs determined in this study.

In agreement with the phylogenetic pattern discerned in the tree of complete mtDNA sequences, all 1,096 mtDNA sequences of East Asian wild boars and domestic pigs could be classified into clade A (Additional data file 5). The wild boars from Island South East Asia reported in a previous study by Larson and coworkers [16] could not be assigned into clades E and A defined in the present study (Additional data file 5 [Table S5]), confirming their basal phylogenetic positions [16,28].

The resulting mtDNA haplogroup classification of wild boars and domestic pigs and their sampling locations revealed sympatric distribution of both wild boars and domestic pigs. Samples from different places/breeds could be grouped into one haplogroup, whereas samples from the same location were assigned to different nested haplogroups (Additional data files 5 and 6, and Tables 1 and 2). Some wild boars from South Asia fell outside of macro-clade containing clades E and A, whereas other wild boars from this region could be classified into clades A and E (Additional data file 4 and 6, and Table 1). Within East and South East Asia, the matrilineal pool of wild boars from the Mekong region contained nearly all the main lineages presented in other regions (Additional data file 6 and Table 1). Furthermore, genetic diversity of wild boars from this region was much greater than that in other regions (except for the upstream region of the Yangtze River [URYZ; Additional data file 7], in which diversity was comparable but this region had a different proportion of matrilineal components). Although high diversity in a region may be caused by influx of haplotypes from different regions, this possibility might be very low here because it also applies to other regions, whereas we failed to observe many centers of diversity.

**Table 1:** Geographic distribution of Asian wild boars

Haplogroup	NEA (48)	UMYR (33)	MDYZ (46)	SC (25)	URYZ (31)	Taiwan (6)	Mekong (59)	SA (12)	Japan (43)	SPI (48)	AN (33)^b^	Total (335)^a^
D1a1a	0	0	14	0	0	0	0	0	0	0	4	14
D1a2	0	0	0	1	0	0	2	0	0	0	0	3
Others in D1a	0	0	0	0	1	0	3	0	0	0	4	4
D1b	0	0	0	0	0	0	2	0	0	0	0	2
D1c	0	0	0	0	0	0	2	0	0	0	0	2
D1d	0	0	3	0	0	0	0	0	0	0	0	3
D1e	0	0	1	0	0	0	1	0	0	0	2	2
D1f	21	0	0	0	0	0	0	0	0	0	0	21
D1h	0	0	0	0	0	0	3	0	0	0	0	3
D1i	0	0	2	0	1	0	0	0	0	0	0	3
D1*	17	0	4	0	0	1	12	0	3	0	9	37
D2	0	0	0	0	2	0	3	0	0	0	0	5
D3	8	2	1	4	0	0	4	0	10	0	9	29
D4	0	0	4	13	2	0	7	0	0	0	0	26
D*	1	0	0	0	0	3	0	0	6	0	0	10
A1a without D	0	0	15	0	2	2	3	0	5	0	0	27
A1 without A1a	1	31	2	4	21	0	9	0	6^c^	0	0	74
A without A1	0	0	0	0	2	0	8	5	0	0	14	15
Others^e^	0	0	0	0	0	0	0	7	13^d^	48	0	55

Note that the samples with unknown location or status as being wild boar or domestic pig are not counted here. The sample size for each geographic region is given in parentheses. ^a^Only wild boars are counted. ^b ^Feral pigs. ^c^Samples from Ryukyu, Japan. ^d^Ancient DNAs. ^e^Sequences that could not be included in clade A.

**Table 2:** Geographic distribution of Asian domestic pigs

Haplogroup		NEA (22)	UMYR (54)	DRYR (33)	URYZ (88)	MDYZ (174)	Mekong (174)	SC (46)	Japan (20)^d^	SA (6)	Other (42)	AN (33)^c^	Total (688)^e^
D1a1a	Individuals (%)^a^	72.7	22.2	72.7	12.5	51.1	13.3	10.9	2.6	0	14.3	12.1	25.8
D1a1a	Haplotypes^b^	4 (2)	1 (0)	0	9 (4)	15 (9)	4 (0)	5(0)	-	0	4 (2)	2 (1)	-
D1a2	Individuals (%)	0	38.9	0	52.3	1.7	13.3	0	0	0	0	0	14.7
D1a2	Haplotypes	0	0	0	6 (2)	1 (0)	10 (4)	0	0	0	0	0	-
D1a3	Individuals (%)	0	14.8	0	0	1.7	0	0	0	0	0	0	1.7
D1a*	Individuals (%)	0	0	0	6.8	7.5	2.2	34.8	0	0	4.8	12.1	6.4
D1b	Individuals (%)	0	0	0	0	0	26.1	0	6.6	0	45.2	0	14.1
D1b	Haplotypes	0	0	0	18.2	1 (0)	3 (1)	0	0	0	3 (0)	0	-
D1c	Individuals (%)	0	13.0	27.3	1.1	5.7	7.2	17.4	0	0	0	0	6.1
D1d	Individuals (%)	0	0	0	0	1.7	0	0	0	0	0	0	4.7
D1e	Individuals (%)	0	0	0	1.1	4.6	0.5	37.0	1.3	0	0	6.1	4.4
D1g	Individuals (%)	0	9.3	0	2.3	0.0	0	0	0	0	0	0	1.1
D1h	Individuals (%)	0	1.9	0	0	0.6	1.1	0	0	0	0	0	0.6
D1i	Individuals (%)	0	0	0	5.6	0	0	0	0	0	0	0	0.8
D1*	Individuals (%)	27.3	0	0	0	23.0	8.3	0	6.6	0	2.4	0	10.5
D2 to D4	Individuals (%)	0	0	0	0	0.6	27.8	0	26.3	66.7	28.6	27.3	13.6
D*	Individuals (%)	0	0	0	0	0	0	0	46.1	0	4.8	0	5.8
A1 without A1a	Individuals (%)	0	0	0	0	0	0	0	10.5	0	0	0	1.3
A without A1	Individuals (%)	0	0	0	0	0	0	0	0	33.3	0	42.4	0.3

The sample size for each geographic region is given in parentheses. ^a^Numbers refer to the proportion of individuals that are grouped into this haplogroup in each region. ^b^Number of haplotypes in each region. The values in parentheses refer to the number of unique haplotypes in that region. We only counted number of haplotypes and unique haplotypes for the three main haplogroups (D1a1a, D1a2, and D1b) that had a large sample size. ^c^Individuals in this column refer to feral pigs. ^d^Ancient DNAs. ^e ^Ancient DNAs and feral pigs are not included.

Most wild boars from the middle and downstream region of the Yangtze River (MDYZ) were clustered into the nested haplogroups within haplogroup A1a, particularly in haplogroup D (Additional data file 6 and Table 1). All wild boars from North East Asia (NEA) belonged to haplogroup D (Additional data file 6 and Table 1), suggesting potential derivation from the matrilineal pool of region MDYZ caused by the natural movement of wild boar. Wild boar lineages from the upstream and middle region of the Yellow River (UMYR) were a subset of region URYZ (Additional data file 6 and Table 1). Overall, the population structure of URYZ and UMYR were distinct from MDYZ and NEA populations; both URYZ and UMYR populations contained more basal lineages, whereas the MDYZ and NEA populations harbored a large proportion of recently derived lineages (Additional data file 6 and Table 1). Under the hypothesis of selective neutrality and population equilibrium, Tajima's D and Fu's *Fs *test values tend to be negative under an excess of recent mutations, which is regarded as evidence of population growth [43,44]. The *P *values of the *Fs *test established by Fu [43] for all wild boar samples belonging to haplogroups D1 and D all indicated statistical significance (Table 3), suggesting population expansion in the past. Taken together, the above haplogroup distribution pattern suggests that East Asian wild boar lineages were most likely derived from the Mekong region population and dispersed via two main routes: one route is through the Yangtze River region to NEA, and the other is through URYZ to UMYR (Additional data file 6). The difference between the current population structures of wild boars in these regions might be shaped by the early dispersal of the wild boars out of the Mekong region.

**Table 3:** Neutrality test and genetic diversity for main haplogroups in East Asian domestic pigs and wild boars

Haplogroup	Domestic or wild	No.	Haplotype diversity	Nucleotide diversity	*Fs *test	Tajima's D
D1a1a	Domestic	291	0.781 ± 0.021	0.00267 ± 0.00016	-18.888*	-1.732*
D1a2	Domestic	98	0.827 ± 0.040	0.00267 ± 0.00021	-27.869*	-1.158
D1a	Domestic	393	0.900 ± 0.008	0.00488 ± 0.00018	-43.161*	-1.824*
D1b	Domestic	88	0.601 ± 0.054	0.00177 ± 0.00013	-5.104*	-1.138
D1c	Domestic	49	0.842 ± 0.022	0.00445 ± 0.00029	-1.538	0.658
D1	Domestic	607	0.943 ± 0.004	0.00556 ± 0.00014	-25.487*	-1.882*
D1	Wild boar	65	0.845 ± 0.051	0.00357 ± 0.00042	-10.64*	-1.07984
D3	Domestic	42^a^	0.573 ± 0.081	0.00233 ± 0.00053	-2.779*	-1.861*
D4	Domestic	18	0.743 ± 0.052	0.00534 ± 0.00054	0.173	0.802
D	Domestic	689	0.954 ± 0.003	0.00691 ± 0.00014	-25.337*	-1.830*
D	Wild boar	136	0.946 ± 0.036	0.00600 ± 0.00039	-25.582*	-0.30877

All of the values were calculated based on mitochondrial DNA (mtDNA) control region sequences. *P *< 0.05 in the neutrality test was regarded as statistically significant (indicated by asterisks [*]). Haplogroup D1a1 contained only one mtDNA besides samples belonging to D1a1a and was not listed. Haplogroups containing five or fewer samples were not analyzed. ^a^Including feral pigs.

Our classification analysis of the published Ryukyu island wild boar samples suggested that none of these samples belonged to haplogroup D, which was dominant in the adjacent regions, such as MDYZ, Taiwan, and Japanese islands (Additional data files 5 and 6). This unique distribution pattern might be attributed to insufficient sampling of Ryukyu island wild boars, or wild boars might have dispersed to Ryukyu Islands before the arrival of haplogroup D and were subsequently isolated from the adjacent regions. This latter scenario is consistent with the suggestion of a different origin of wild boars in Japanese islands and Ryukyu islands [45].

**Figure 2:**
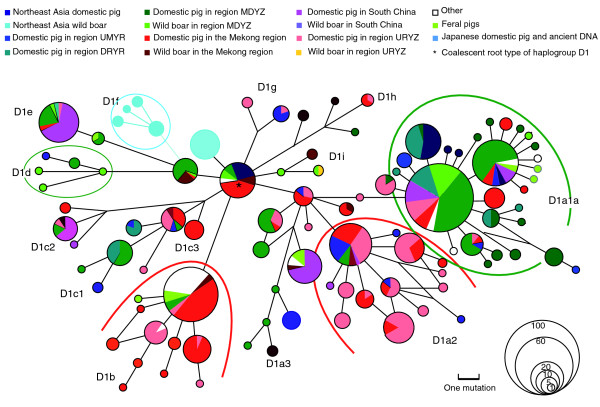
Reduced median network. Shown is a reduced median network of domestic pig and wild boar mitochondrial DNAs (mtDNAs) belonging to haplogroup D1 based on the sequence variation of control region and coding region fragments. The mtDNA control region fragment covers the region from 1 to 670, and the coding region fragments cover regions 1860 to 2400, 3030 to 3800, 3940 to 4520, 4730 to 5340, 5530 to 6175, 6410 to 7920, 8045 to 11470, 12050 to 12675, 14121 to 14617, and 14730 to 16176 relative to the reference sequence EF545567. These samples are from the Mekong region, the upstream region of the Yangtze River (URYZ), the middle and downstream region of the Yangtze River (MDYZ), South China (SC), the upstream and middle region of the Yellow River (UMYR), the downstream region of the Yellow River (DRYR), North East Asia (NEA), Japan, Australia, New Zealand, and other places. Each haplotype is represented by a circle, with the area of the circle proportional to its frequency. The haplotype with an asterisk is the coalescent root type of D1. Samples from different regions were indicated by different colors. The length of each branch is proportional to the number of mutations on the respective branch.

In the phylogenetic tree of complete mtDNA sequences, all East Asian domestic pig mtDNAs were clustered into single subclade D, with wild boars from this region interspersed (Figure 1 and Additional data files 3 and 4). The haplogroup classification of all available East Asian domestic pigs also uniformly referred to haplogroup D, which contains four subhaplogroups: D1, D2, D3, and D4. However, only part of wild boar samples in this region could be allocated to haplogroup D (Additional data files 5 and 6, and Table 1). This pattern suggests that East Asian domestic pigs originated from a subset of the wild boar genetic pool that was characterized by haplogroup D. Direct comparison of the geographic distribution between wild boars and domestic pigs can provide clues regarding the domestication of East Asian pigs. All domestic pig samples from regions NEA, URYZ, UMYR, and the downstream region of the Yellow River (DRYR), clustered within haplogroup D1 (Additional data file 6 and Figure 2). The wild boars from region NEA belonged to haplogroups D3 and D1f (excluding one unique yet unassigned D1 haplotype because of absence of coding region information), and did not share any haplotype with all of the domestic pigs from this region (Figure 2, Tables 1 and 2, and Additional data files 5 and 6). None of the 32 wild boars from region UMYR belonged to haplogroup D1 (Additional data file 6 and Table 1), although archeologic assemblages from this region exhibit signs of pig domestication during the Neolithic period [46]. Among the 32 wild boars from region URYZ, only one individual could be assigned to D1 (Additional data file 6 and Table 1). In contrast, the wild boars in the Mekong region and region MDYZ extensively shared haplotypes with domestic pigs that were clustered into different haplogroups (Figure 2 and Additional data files 5 and 6): haplogroups D2, D3, D4, D1a2, D1b, and D1c contained both wild boars and domestic pigs from the Mekong region; and haplogroups D1a1a, D1d, and D1e contained both wild boars and domestic pigs from region MDYZ. These distinct phylogeographic patterns of wild boars and domestic pigs indicate that domestication events might have occurred mainly in the Mekong region and the MDYZ region.

The limited regional distributions of some haplogroups in domestic pigs, such as D2, D3, and D4, would suggest *in situ *domestication (Additional data file 6 and Table 2). Domestic pigs belonging to haplogroups D2, D3, and D4 were mainly found in the Mekong region, whereas only a small portion of domestic pigs (three samples) belonging to these three haplogroups was found in region MDYZ. Furthermore, wild boars from the Mekong region harbored lineages belonging to all three haplogroups, whereas wild boars from other places only contained one or two of the three haplogroups (Additional data file 6). None of 12 South Asian wild boars from a previous study [16] could be assigned to haplogroup A1 (including its subhaplogroups), suggesting that they made no contribution to domestic pigs in haplogroups D2, D3, and D4. Four out of six Indian domestic samples belonged to haplogroups D2 and D3 (the other two domestic samples had same A* status as wild boars from this region and the one reported by Larson and coworkers [16] shared an A* haplotype with the wild boars from this region; see Additional data file 5 [Tables S1 and S6]); and nine Australian feral pigs, seven New Zealand domestic pigs, and three European domestic pigs could be classified into D2, D3, and D4. It is possible that these lineages were derived from the same matrilineal pool with the domestic pigs from the Mekong region (Additional data file 6). The distinguished regional distribution pattern of haplogroups D2, D3, and D4 in domestic pigs and wild boars suggested that they probably originated from the Mekong region and/or adjacent regions that we did not sample in the present study.

Haplogroup D1 harbors more than 90% of the domestic animals, which were widely distributed in various regions in East Asia (Table 2, and Additional data files 5 and 6). The regional distribution of the main founders belonging to this haplogroup was depicted in a reduced median network (Figure 2). The main subhaplogroups in haplogroup D1 were almost equidistant to their coalescent ancestral root type. Some of them, such as haplogroups D1a1a, D1a2, and D1b, exhibited a star-like profile that was typical of exponential population growth. Each of these haplogroups harbored one or two widely distributed major haplotypes, which were also found in wild boars and had many one-mutation or two-mutation distance derivatives that were detected exclusively in domestic pigs. Our estimations for domestic pigs in the major haplogroups within D all revealed negative Tajima's D and Fu's *Fs *test values (not including D4 and D1c). The *P *values of the *Fs *test indicated statistical significance for haplogroups D1a1a (Tajima's D test was also statistically significant for this haplogroup), D1a2, and D1b, suggesting potential population expansion in the past (Table 3). Taken together, haplotypes within each of these haplogroups might have originated from their major (central) haplotypes as results of domestication events followed by subsequent expansion. Tracing the geographic distribution pattern of these haplogroups might reveal more information about the domestication events, as discussed below.

Most of the domestic pigs in haplogroups D1a2 and D1b were from the Mekong region and the URYZ region, whereas wild boars in these two haplogroups were exclusively from the Mekong region (Additional data file 6, Figure 2, and Tables 1 and 2). A small portion of domestic pigs (18.5%) in region UMYR belonged to D1a2 and shared haplotypes (excluding two D1a2 individuals) with samples from the Mekong region. However, there are only a few domestic pigs from region MDYZ in haplogroups D1a2 and D1b (three D1a2 types and three D1b types; all sharing haplotypes with the samples from the Mekong region). None of the domestic pigs in the other regions, such as South China (SC), region DRYR, and region NEA, belonged to D1a2 and D1b. This unique genetic pattern of haplogroups D1a2 and D1b suggested that they might have originated in the Mekong region and then dispersed northward to regions URYZ and UMYR (Additional data file 6 and Figure 2). The shared D1a2 and D1b mtDNA types between the MDYZ region and the Mekong region might have been introduced from the Mekong region after the initial domestication.

More than half of the domestic pigs in haplogroup D1a1a were from regions DRYR and MDYZ (Additional data file 6, Figure 2, and Table 2). Fourteen wild boars in haplogroup D1a1a were only found in region MDYZ. Domestic pigs in region MDYZ also possessed a greater number of D1a1a haplotypes and unique haplotypes than did samples from other regions (Table 2). Thus, the D1a1a domestic pigs might have originated from the wild boar population in region MDYZ, which was regarded as one of the origin and dispersal centers of cultivated rice and the agriculture civilization of East Asia [47-49]. Most of the domestic individuals from regions NEA and DRYR shared haplotypes with pigs from region MDYZ, which suggested that domestic pigs from these two regions were most likely derived from the MDYZ pool (Additional data file 6 and Figure 2).

By reanalyzing them with previously reported data, the new data generated in the present study could yield some valuable insights into pig origin in Japan and Vietnam. Pig husbandry was interrupted from the 8th century to the late 19th century on mainland Japan [50]. Evidence of the origin of Japanese domestic pigs was mainly estimated from cultural records and ancient DNA studies. Recent studies of ancient DNA conducted in pig and wild boar remains from the Japanese mainland and islands suggested that Japanese domestic pigs were introduced from China [15,50,51].

Based on the haplogroup classification system established in this study, the Sakhalin pig ancient DNAs from the Kabukai A site (centuries 5 to 8 AD) of the Okhotsk cultural area [51] and the ancient DNAs from the Jomon period (6,100 to 1,700 years old) [15] could be classified: 16 haplotypes fell outside haplogroup D; ten haplotypes belonged to haplogroupd D but could not be assigned into its defined subhaplogroups; and five, one, and four haplotypes belonged to haplogroups D3, D4, and D1, respectively (Additional data file 5 [Table S3]). None of these ancient DNAs shared haplotypes with East Asian domestic pigs (excluding one sequence that shared a haplotype with domestic pigs; Additional data file 6 and Additional data file 5 [Table S3]). Similar matrilineal components were also found among local wild boars (Additional data file 6). Therefore, most of these ancient DNAs were more closely related to local and North East Asia wild boars than to East Asia domestic pigs. Ancient DNA of *Sus scrofa *specimens from Ryukyu Shimizu shell midden (Yayoi-Heian period; 1,700 to 2,000 before present) [50] and Ryukyu wild boars belonged to haplogroup A1b, which diverged earlier than haplogroup D (Table 1 and Additional data file 6). It is thus clear that these ancient DNA might not be the domestic pigs introduced from the Asian continent in the early Yayoi-Heian period. More archaeologic evidence and genetic data from Japan and its adjacent continental regions are necessary to refine further the origin of Japanese domestic pigs.

A previous study of pigs in Vietnam showed that large Vietnamese pigs were wild boars and had close genetic affinity to Ryukyu wild boars, whereas small Vietnamese pigs were domestic pigs and closely related to East Asian domestic pigs, suggesting a local domestication or direct introduction from Southwest China [52]. Reanalysis of these data showed that large Vietnamese pigs shared the same lineage (A1b) with the Ryukyu wild boars and wild boars from the Mekong region, URYZ, and UMYR (Additional data file 5 [Table S1]). Among the haplotypes identified in small Vietnamese pigs, one haplotype belonged to D1a1a and the other haplotypes belonged to haplogroups presented in the Mekong region, such as D3 and D1b (Additional data file 5 [Table S1]). Six haplotypes were also found in small pigs from Yunnan, China, and pigs from Laos (Additional data file 5 [Table S1]). Our reanalysis of these Vietnamese pig mtDNAs further demonstrated that large pigs and small pigs from this region had different matrilineal components; thus, in general it supports the previous claim that small Vietnamese pigs were introduced from China and the Mekong region, whereas large Vietnamese pigs were local wild boars [52].

## Conclusion

In the present study, use of a phylogeny of complete mtDNA sequences allowed us to conduct a fine-grained phylogeographic analysis of the Asian domestic pigs and wild boars and to reappraise the published data. This approach could also be utilized to elucidatde the origin of other domestic animals such as chicken, cattle, sheep, and goats. Our findings indicate that the current domestic pig regional pools in East Asia originated from a subset of wild boar matrilineal components belonging to haplogroup D. These major matrilineal components in domestic pigs, such as D2, D3, D4, D1b, and D1a2, were probably domesticated in the Mekong region. Region MDYZ might also be a domestication center for lineages in D1a1a, D1d, and D1e. The initial domesticated pool was composed of a number of founders (at least ten) and underwent subsequent northward dispersal but with limited admixture.

## Materials and methods

### Samples

In total, 567 domestic pigs and 155 wild boars from China, South East Asia, and India were collected and analyzed for mtDNA control region sequence variation. Among them, 24 samples (not including one African warthog [*Phacochoerus africanus*] and a wild boar sample from Maylasia [*Sus barbatus*]; Additional data file 2) were selected for complete mtDNA sequencing. The published pig and wild boar mtDNA sequences in Asia [5,15,16,26,28,40,41,45,51-61] were retrieved from GenBank and were reanalyzed (Additional data file 5). The ancient DNA sequences from Japanese islands [15,51] were only scored according to the haplotype information because the precise number of individuals sharing a haplotype was not listed in the original reports or GenBank deposits.

### DNA extraction, PCR amplification, and sequencing

Genomic DNA was extracted from whole blood, tissue, and/or hair using the standard phenol/chloroform method. The mtDNA control region sequence (670 bp) was amplified using primer pair H695 (5'-CTCTTGCTCCACCATCAGC-3') and L99 (5'-AAACTATATGTCCTGAAACC-3'). Complete mtDNA sequences were amplified and sequenced using different combinations of 36 to 40 pairs of primers (Additional data file 8). Polymerase chain reaction (PCR) products were purified on spin columns (Watson BioTechnologies, Shanghai, China) and sequenced by using BigDye Terminator sequencing kit (Applied Biosystems, Foster City, California, USA). Sequencing was performed on a 377 and 3700 DNA sequencer (Applied Biosystems). Sequences were edited by using the DNASTAR software (DNAstar Inc. Madison, Wisconsin, USA) and mutations were scored relative to a reference sequence (individual Saba722; accession number EF545567) determined in the present study. All mtDNA D-loop and complete genome sequences have been submitted to GenBank (accession numbers DQ409327, DQ496251 to DQ497000, and EF545567 to EF545593).

### Phylogenetic analysis

An unrooted NJ tree was initially constructed based on the haplotypes (670 bp fragments) in all 722 samples. Twenty-four samples of wild boars and domestic pigs, each representing a unique haplotype from the major clades in the NJ tree, were selected for complete mtDNA sequencing. An African warthog was sequenced and used as the outgroup for phylogenetic analyses of the complete mtDNA sequences. The phylogenetic consensus tree of 50 mtDNA complete and near complete sequences (one warthog, one Malaysia wild boar, six European pigs and wild boars [note that sequences AF034253 and NC_000845 [56] should refer to the same sample], and 42 Asian pigs and wild boars; Additional data file 2) was constructed by using the NJ method in Phylogenetic Analysis Using Parsimony (PAUP) 4.0 β [62] with the model of HKY + I + G (shape α = 1.0714; Pinvar = 0.7512), as recommended by Modeltest 3.6 [63]. The maximum parsimony tree of these mtDNA sequences was constructed by using the branch-and-bound search with a tree bisection-reconnection (TBR) branch-swapping option in PAUP. Robustness of the nodes was assessed by the bootstrap method after 1,000 replications (bootstrap option with heuristic search in PAUP) by adding sequences randomly. Bayesian inference tree was constructed by MrBayes 3.1 [64] with the general time reversible (GTR) model. In an initial run, the likelihood of the cold chain stopped increasing and began to fluctuate randomly within a more or less stable range after 10,000 generations; this suggests that the run may have reached stationarity. Three independent runs (each with 1 million generations) were performed. Each run was started from a randomly chosen but different tree. All of these runs yielded similar estimates of substitution model parameters, topology, and branch lengths (Additional data file 4).

### Haplogroup classification

We denoted the principal clades (or haplogroups) that emerged in the phylogenetic tree by capital letters (for example, clade E [European] and clade A [Asian]). For the prominent clade containing all East Asian domestic pigs and some wild boars, we designated it by the capital letter D. For other subclades of clade A and the subclades within D, a hierarchical haplogroup nomination system was used, as for human mtDNA [30,32-34]. Thus, the code signifies the nested haplogroup relationships (for example, D1a1a ⊂ D1a1 ⊂ D1a ⊂ D1⊂ D ⊂ A1a ⊂ A1 ⊂ A.

Each haplogroup was composed of a cohort mtDNAs that shared a string of characteristic mutations, which could be read from the complete mtDNA tree [29,30,32,36,37] (Additional data file 3). We screened the haplogroup-specific mutations in all of our samples to justify the haplogroup assignment of each sample. If a mtDNA could be assigned to a haplogroup but could not be further assigned to its specific subhaplogroups, then an asterisk (*) is attached to the haplogroup name that refers to the mtDNA under consideration, in order to emphasize that the haplogroup status of the mtDNA cannot be specified further (relative to the classification tree) [33,34]. For example, haplogroup A has two named subhaplogroups A1 and A2, and the Indian wild boars could be assigned to haplogroup A based on the available sequence variation information, but they could not be further assigned to A1 or A2 and lacked the necessary mtDNA coding region information to identify a new haplogroup nested in A. Therefore, they were left unassigned and denoted A*. Thus, A* contains all mtDNAs that were grouped into A but fell outside A1 and A2 (A* = A - A1 - A2).

After each mtDNA was classified into its respective haplogroup (Additional data file 5), the haplogroup distribution frequency in each geographic region (see below) was estimated. The published pig mtDNA Cyt *b *sequences [5,45] were tentatively assigned to haplogroups according to the established classification system. The reported partial mtDNA D-loop sequences [5,15,16,26,40,41,45,51-55,58,59,65] were also classified by a matching or near matching strategy with the mtDNAs determined in this study as well as by mutation motif recognition, as described in human mtDNA studies [30,34,35,38].

### Geographic group classification

We grouped the samples into the following groups according to geographic fauna and possible pig domestication sites (Additional data file 6 and Figure 2). The Mekong region includes northwest, south and southeast Yunnan, China, Burma, Laos, north Vietnam, and north Thailand. Region URYZ includes Sichuan, Chongqing, Guizhou, north and northeast Yunnan, northwest Guangxi, west Hebei, and northwest Hunan. Region MDYZ includes east Hubei, northeast Hunan, Anhui, Jiangxi, Fujian, Zhejiang, Jiangsu, and Shanghai. Fourth, region SC includes Guangdong, south and southeast Guangxi, south Hunan, southwest Fujian, and Hainan. Region UMYR includes Gansu, east Qinghai, northwest Sichuan, south Inner Mongolia, Ningxia, Shaanxi, Shanxi, and west Henan. Region DRYR includes east Henan, Hebei, and Shandong. Region NEA includes Jilin, Liaoning, Heilongjiang, northeast Inner Mongolia, southeast Siberia, and Korea. Region SPI (South Pacific Islands) includes South Pacific Islands and the Malay Peninsula. Region AN includes feral pigs in Australia and New Zealand. 'Other' includes domestic pigs with Asian mtDNA type found in Europe, Australia, New Zealand, and America.

### Network construction

To provide more detailed information on the phylogeographic relationship among the wild boars and domestic pigs belonging to haplogroup D1, which contained most of the samples analyzed in this study, a reduced median network [66] was constructed by using Network 4.1 [67].

### Estimation of population expansion

Tajima's D test [44] and Fu's *Fs *test [43] was employed to test whether neutrality holds (the population under study evolves with a constant effective population size, all mutations being selectively neutral) by using Arlequin 3.1 [68]. A population that has experienced population expansion may result in a rejection of the null hypothesis. We also estimated the haplotype diversity (h) and nucleotide diversity (π) [69] for main haplogroups nested in D1 using DnaSP 4.0 [70].

## Abbreviations

bp, base pair; Cyt, cytochrome; DRYR, downstream region of the Yellow River; MDYZ, middle and downstream region of the Yangtze River; mtDNA, mitochondrial DNA; NEA, North East Asia; NJ, neighbor-joining; PAUP = Phylogenetic Analysis Using Parsimony; PCR, polymerase chain reaction; SC, South China; UMYR, upstream and middle region of the Yellow River; URYZ, upstream region of the Yangtze River.

## Authors' contributions

YPZ, GSW, and YGY conceived and designed the experiments. GSW, KXQ, ZLD, and HL performed the experiments. GSW, YGY, and YPZ analyzed the data. GSW, MGP, ZYD, NL, and YSC collected samples. GSW, YGY, and YPZ wrote the paper. All authors read and approved the final manuscript.

## Additional data files

The following additional data are available with the online version of this paper. Additional data file 1 shows an unrooted NJ tree of 119 mtDNA D-loop sequence haplotypes identified in 722 wild boar and domestic pig samples. Additional data file 2 provides the sample information for the complete mtDNAs analyzed in this study. Additional data file 3 shows the classification tree of 42 (near) complete mtDNA sequences in clade A in Figure 1. Additional data file 4 shows the phylogenetic trees constructed using the maximum parsimony and the Bayesian methods. Additional data file 5 contains six tables (listing mtDNA sequence information and haplogroup classification of wild boars and domestic pigs analyzed in this study. Additional data file 6 shows the phylogeographic distribution of haplogroups and hypothetical dispersal routes of East Asian wild boars and domestic pigs. Additional data file 7 is a table showing genetic diversity of samples in each geographic region. Additional data file 8 is a table listing all of the primers used for pig complete mtDNA sequencing and haplogroup motif detection.

## Supplementary Material

Additional data file 1 Unrooted NJ tree. Shown is an unrooted NJ tree of 119 mtDNA D-loop sequence haplotypes identified in 722 wild boar and domestic pig samples. Click here for file

Additional data file 2 Sample information for the complete mtDNAs. Presented is sample information for the complete mtDNAs analyzed in this study. Click here for file

Additional data file 3 Classification tree of 42 (near) complete mtDNA sequences in clade A. Shown is the classification tree of 42 (near) complete mtDNA sequences in clade A in Figure 1. Click here for file

Additional data file 4 Phylogenetic trees: maximum parsimony and the Bayesian methods. Presented are phylogenetic trees calculated using the maximum parsimony and the Bayesian methods. Click here for file

Additional data file 5 mtDNA sequence information and haplogroup classification. Presented are six tables (Tables S1 to S6) listing mtDNA sequence information and haplogroup classification of wild boars and domestic pigs analyzed in this study. Click here for file

Additional data file 6 Phylogeographic distribution of haplogroups and hypothetical dispersal routes. Presened are phylogeographic distribution of haplogroups and hypothetical dispersal routes of East Asian wild boars and domestic pigs. Click here for file

Additional data file 7 Genetic diversity of samples. Presented is a table showing genetic diversity of samples in each geographic region. Click here for file

Additional data file 8 Primers. Presented is a table listing all the primers used for pig complete mtDNA sequencing and haplogroup motif detection. Click here for file
