# Supplementary material for: Population phylogenomic analysis of mitochondrial DNA in wild boars and domestic pigs revealed multiple domestication events in East Asia
Source: Genome Biol. 2007 Nov 19;8(11):R245. doi: 10.1186/gb-2007-8-11-r245 (PMC2258183; doi:10.1186/gb-2007-8-11-r245)
Supplement: Additional data file 1 — Unrooted NJ tree. Shown is an unrooted NJ tree of 119 mtDNA D-loop sequence haplotypes identified in 722 wild boar and domestic pig samples. [file gb-2007-8-11-r245-S1.doc]

**Additional data file 1**. Unrooted neighbor-joining tree of mtDNA D-loop sequence haplotypes identified in 722 wild boar and domestic pig samples
