# Supplementary material for: Population phylogenomic analysis of mitochondrial DNA in wild boars and domestic pigs revealed multiple domestication events in East Asia
Source: Genome Biol. 2007 Nov 19;8(11):R245. doi: 10.1186/gb-2007-8-11-r245 (PMC2258183; doi:10.1186/gb-2007-8-11-r245)
Supplement: Additional data file 3 — Classification tree of 42 (near) complete mtDNA sequences in clade A. Shown is the classification tree of 42 (near) complete mtDNA sequences in clade A in Figure 1. [file gb-2007-8-11-r245-S3.ppt]

## Slide 1
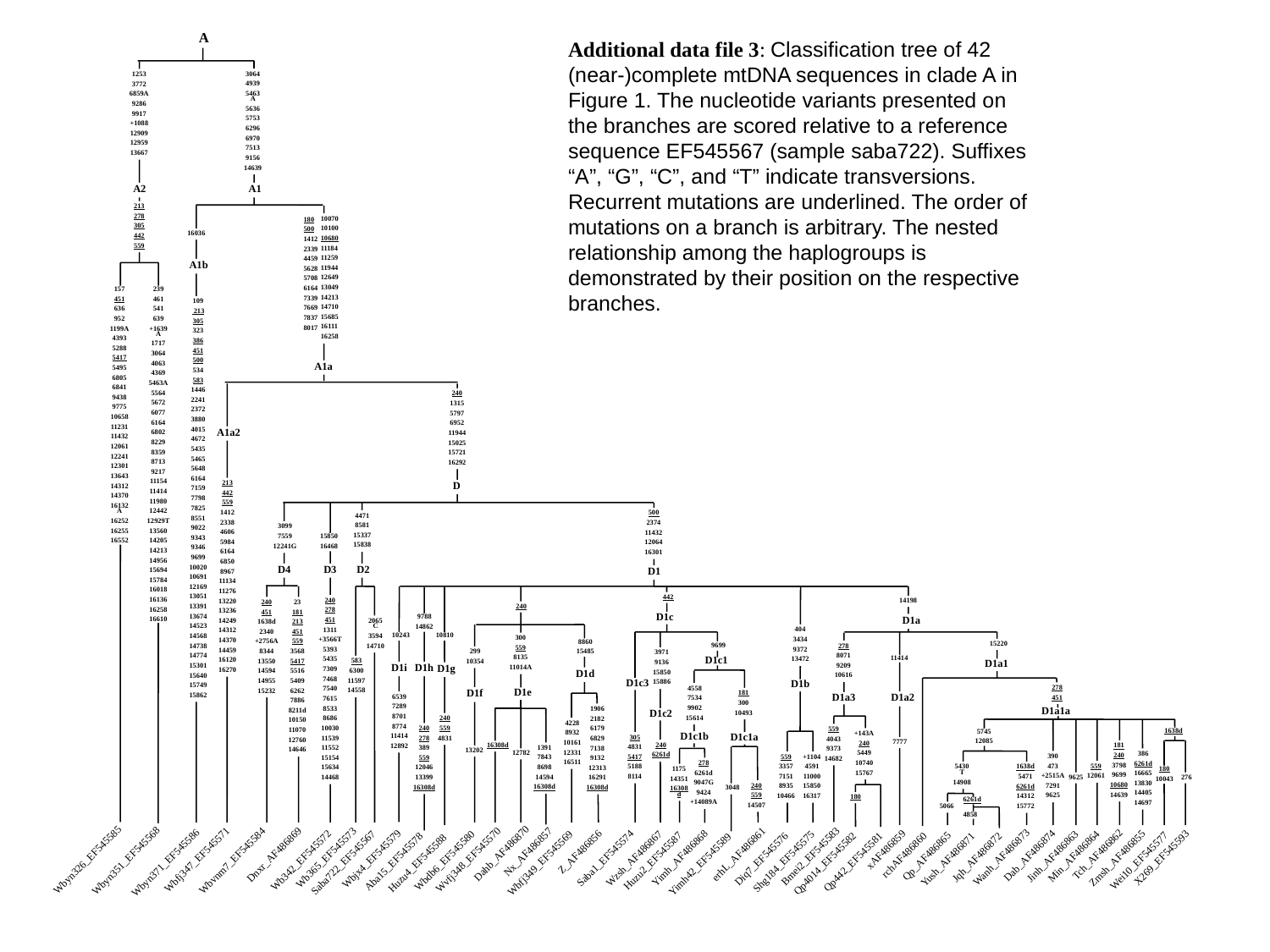

Additional data file 3: Classification tree of 42 (near-)complete mtDNA sequences in clade A in Figure 1. The nucleotide variants presented on the branches are scored relative to a reference sequence EF545567 (sample saba722). Suffixes “A”, “G”, “C”, and “T” indicate transversions. Recurrent mutations are underlined. The order of mutations on a branch is arbitrary. The nested relationship among the haplogroups is demonstrated by their position on the respective branches.
A
3064
4939
5463A
5636
5753
6296
6970
7513
9156
14639
1253
3772
6859A
9286
9917
+1088
12909
12959
13667
A2
A1
213
278
305
442
559
10070
10100
10680
11184
11259
11944
12649
13049
14213
14710
15685
16111
16258
180
500
1412
2339
4459
5628
5708
6164
7339
7669
7837
8017
16036
A1b
157
451
636
952
1199A
4393
5288
5417
5495
6805
6841
9438
9775
10658
11231
11432
12061
12241
12301
13643
14312
14370
16132A
16252
16255
16552
239
461
541
639
+1639A
1717
3064
4063
4369
5463A
5564
5672
6077
6164
6802
8229
8359
8713
9217
11154
11414
11980
12442
12929T
13560
14205
14213
14956
15694
15784
16018
16136
16258
16610
109
 213
305
323
386
451
500
534
583
1446
2241
2372
3880
4015
4672
5435
5465
5648
6164
7159
7798
7825
8551
9022
9343
9346
9699
10020
10691
12169
13051
13391
13674
14523
14568
14738
14774
15301
15640
15749
15862
A1a
240
1315
5797
6952
11944
15025
15721
16292
A1a2
213
442
559
1412
2338
4606
5984
6164
6850
8967
11134
11276
13220
13236
14249
14312
14370
14459
16120
16270
D
500
2374
11432
12064
16301
4471
8581
15337
15838
3099
7559
12241G
15850
16468
D4
D3
D2
D1
442
240
278
451
1311
+3566T
5393
5435
7309
7468
7540
7615
8533
8686
10030
11539
11552
15154
15634
14468
14198
240
451
1638d
2340
+2756A
8344
13550
14594
14955
15232
23
181
213
451
559
3568
5417
5516
5409
6262
7886
8211d
10150
11070
12760
14646
240
D1c
9788
14862
D1a
2065C
3594
14710
404
3434
9372
13472
10243
10810
300
559
8135
11014A
8860
15485
15220
278
8071
9209
10616
9699
299
10354
3971
9136
15850
15886
11414
583
6300
11597
14558
D1c1
D1a1
D1i
D1h
D1g
D1d
D1c3
D1b
278
451
4558
7534
9902
15614
D1f
D1e
181
300
10493
6539
7289
8701
8774
11414
12892
D1a2
D1a3
1906
2182
6179
6829
7138
9132
12313
16291
16308d
D1a1a
D1c2
240
559
4831
4228
8932
10161
12331
16511
240
278
389
559
12046
13399
16308d
559
4043
9373
14682
1638d
+143A
240
5449
10740
15767
5745
12085
D1c1b
305
4831
5417
5188
8114
D1c1a
7777
240
6261d
181
240
3798
9699
10680
14639
16308d
1391
7843
8698
14594
16308d
13202
12782
386
6261d
16665
13830
14405
14697
390
473
+2515A
7291
9625
559
3357
7151
8935
10466
+1104
4591
11000
15850
16317
278
6261d
9047G
9424
+14089A
559
12061
5430T
14908
1638d
5471
6261d
14312
15772
1175
14351
16308d
180
10043
9625
276
240
559
14507
3048
180
6261d
5066
4858
xAF486859
Z_AF486856
Nx_AF486857
rchAF486860
Tch_AF486862
Qp_AF486865
Dnxr_AF486869
Dahb_AF486870
erhL_AF486861
Dab_AF486874
Jqh_AF486872
Jinh_AF486863
Min_AF486864
Wanh_AF486873
Yimh_AF486868
Zmsh_AF486855
Wzsh_AF486867
Yush_AF486871
Bmei2_EF545583
X269_EF545593
Wbfj347_EF545571
Saba1_EF545574
Wbjx4_EF545579
Diq7_EF545576
Wei10_EF545577
Qp442_EF545581
Wbdb6_EF545580
Huzu2_EF545587
Wb365_EF545573
Aba15_EF545578
Huzu4_EF545588
Wvfj348_EF545570
Wb342_EF545572
Wbyn371_EF545586
Qp4014_EF545582
Shg184_EF545575
Wbyn326_EF545585
Wbyn351_EF545568
Saba722_EF545567
Wbvnm7_EF545584
Yimh42_EF545589
Wbfj349_EF545569
