# Supplementary material for: Population phylogenomic analysis of mitochondrial DNA in wild boars and domestic pigs revealed multiple domestication events in East Asia
Source: Genome Biol. 2007 Nov 19;8(11):R245. doi: 10.1186/gb-2007-8-11-r245 (PMC2258183; doi:10.1186/gb-2007-8-11-r245)
Supplement: Additional data file 4 — Phylogenetic trees: maximum parsimony and the Bayesian methods. Presented are phylogenetic trees calculated using the maximum parsimony and the Bayesian methods. [file gb-2007-8-11-r245-S4.doc]

**Additional data file 4**. Maximum parsimony (Figure S1) and Bayesian trees (Figures S2a, S2b, and S2c) of wild boars and domestic pigs from Asia and Europe based on complete and near-complete mtDNA sequences.

Figure S1. Condensed most parsimonious consensus tree of pig complete mtDNA sequences.

Figure S2a. The Bayesian consensus tree of pig complete mtDNA sequences based on the first independent run.

Figure S2b. The Bayesian consensus tree of pig complete mtDNA sequences based on the second independent run.

Figure S2c. The Bayesian consensus tree of pig complete mtDNA sequences based on the third independent run.
