# Supplementary material for: Population phylogenomic analysis of mitochondrial DNA in wild boars and domestic pigs revealed multiple domestication events in East Asia
Source: Genome Biol. 2007 Nov 19;8(11):R245. doi: 10.1186/gb-2007-8-11-r245 (PMC2258183; doi:10.1186/gb-2007-8-11-r245)
Supplement: Additional data file 6 — Phylogeographic distribution of haplogroups and hypothetical dispersal routes. Presened are phylogeographic distribution of haplogroups and hypothetical dispersal routes of East Asian wild boars and domestic pigs. [file gb-2007-8-11-r245-S6.doc]

**Additional data file 6**. Phylogeographic distribution of haplogroups and hypothetical dispersal routes of East Asian wild boars and domestic pigs. The phylogenetic relationship among the haplogroups is depicted in Figures 1 and 2. The red squares represent wild boars and green squares represent domestic pigs or feral pigs. Ancient DNAs from Japanese Islands and Ryukyu Islands are denoted by pink squares. The area of the square is proportional to the frequency of the respective haplogroup. The speculated dispersal routes of wild boars (red) and domestic pigs (green) were depicted by arrows marked with corresponding color. The geographic region was defined in the Materials and Methods section.
