# Supplementary material for: Population phylogenomic analysis of mitochondrial DNA in wild boars and domestic pigs revealed multiple domestication events in East Asia
Source: Genome Biol. 2007 Nov 19;8(11):R245. doi: 10.1186/gb-2007-8-11-r245 (PMC2258183; doi:10.1186/gb-2007-8-11-r245)
Supplement: Additional data file 8 — Primers. Presented is a table listing all the primers used for pig complete mtDNA sequencing and haplogroup motif detection. [file gb-2007-8-11-r245-S8.doc]

**Additional data file 8**. Primers for pig complete mtDNA sequencing and haplogroup motif detection

| Primer | Sequence (5’-3’) | Primer | Sequence (5’-3’) |
| --- | --- | --- | --- |
| L99 | CCC AAA GCT GAA ATT CTA ACT AAA | H 451 | GGT GAG ATG GCC CTG AAG TAA G |
| L331 | CCG CGT GAA ACC AGC AAC C | H695 | TCC CGT AAC CAT TGA CTG AA |
| L590 | CGT CAA AGG CCC TAA CAC AGT CA | H1048 | TAT TCA GAT TGT GGG CGT ATG CT |
| L1715 | TAA CAA AAC TAT TCG CCA GA | H913 | TGT GTA GAG CAT AAG TTT AA |
| L1756 | TAA AAC TCA AAG GAC TTG GC | H1173 | TAA GCT ATA TTC AGA TTG TG |
| A-L838 | ACA CGC GCA TAT AAG CAG GTA A | AS-H1493 | GGC TGG CAC GAG ATT TAC CA |
| L1919 | ATA TAC CGC CAT CTT CAG C | H1225 | TCA TCT AGG CAT TTT CAG TG |
| AS-L1310 | CCG GTG AGA ATG CCC TCC AG | A-H1888 | CCT TTT TAG GGT TTG CTG AA |
| B-L1729 | CGC CAG AGT ACT ACT CGC AA | BS-H2358 | CTC CTA TAC TTT AAT GTT AG |
| 2-L2216 | GCT TAA ACT AAA GCA CCT AGT | 1-H2395 | GGT ACT ATC TCT ATA GCG TCA |
| L2227 | AGC ACC TAG TTT ACA CCT AGA AG | H2704 | AGC TGT ACC TTT TTA GAC TAT C |
| BS-L2167 | CGT AAC AAG GTA AGC ATA CT | B-H2750 | GTC AAG GTT GTA TCC GTT TC |
| C-L2607 | GCC TAA CGA GCC TGG TGA TA | CS-H3200 | CCT TTG CAC GGT CAG AAT AC |
| L2703 | AGA TAG TCT AAA AAG GTA CAG C | H3126 | TGC CTC TAA TAC TAG TAA TGC |
| L2928 | AAG CCT TTC TCC TCG CAC ACG | 2-H3744 | CTC CTT TCG TAC TGG GAG AAA T |
| CS-L3033 | CCC AAC ACA GGA GTG CAC CA | C-H3597 | GGT CGT AAA CCC TAT TGT CG |
| D-L3453 | CCG AGT GAT TTT AAT CTA GAC | DS-H4034 | TGG GTC CTT TTC GTA GTT GC |
| 3-L3585 | AGG GTT TAC GAC CTC GAT GTT | 3-H5119 | ATG ATA AGG GTA GTG TAG ATA |
| L3844 | ACA TTA GGG TGG CAG AGA C | H4242 | ACT GCT AGG C TTGAC ATG G |
| DS-L3853 | TGG CAG AGA CCG GTA ATT GC | D-H4465 | TAG GGG TCA GGA TGT AAA GA |
| E-L4290 | CCA ACT CAA AAT ACG CAC TC | ES-H4820 | GAG CTA GTG TTA GGG GCA GG |
| L4631 | ATT CAC AGC AAT TCT CTT CC | 4-H6496 | GTA AGC ATT AGA CTG TAA ATC T |
| 4-L4897 | GAG TTA CTT TGA TAG AGT AAA | E-H5288 | GGA GGC TGT GGC TTG TGT TAG G |
| ES-L4660 | CCA CGA CCC ACA CAC ACC AG | H5912 | TTG CTA TGA GTG TTG GCA TG |
| F-L5155 | GCT CAC ACT GAC TAC TCA TC | 4S-H5539 | GGG TTG ATT GAT TGT GAG ATT |
| L5407 | GAC TCT CAC CTT TCC ACT TCT G | 4S-H6051 | GTT TTG TGT GTT CGA ATT GT C |
| 4S-L5406 | GGA CTC TCA CCT TTC CAC TTC | FS-H5767 | GTT GTG GTT GCT GAG CTG TGG |
| 4S-L5845 | GCC TGC CTC CAC TAT CAG GAT | L5954 | CCT CTA TTT CTA CAT ACG AC |
| FS-L5640 | GCA CAC ATA GGA TGA ATG ACA GC | F-H6123 | GGG ACG AGA GGG CTG GTG TT |
| G-L5970 | CGA CTA GCC TAC TCC TCC TC | GS-H6563 | GGG TGC CGA TGT CTT TGT GG |
| 5-L6294 | CCC ACG AAA CTT TTA GTT AAC | H6389 | AAA GAA GCA GCT TCA ATT CTG |
| GS-L6384 | CCC GGC AGA ATT GAA GCT GC | G-H6973 | ACC TGC AAG GTG TAG GGA GA |
| L6774 | GAG CTC CCG ATA TGG CCT TTC | HS-H7393 | GGT GAA TAT GTG GTG AGC CC |
| 6-L7703 | CC CCT ATT CTC CGG GTA CAC A | H-L6850 | GGC ATC CTC AAT AGT AGA AGC |
| L6968 | GCA GGT GTA TCA TCA ATC CTA | HS-L7264 | CGG AAT AAT CTC CCA CAT TG |
| L7194 | CTA TCC TTT ATC AAC ACT TG | 5-H7863 | TTT CAT GCT GTG TAT GCG TCA |
| I-L7713 | CCG GGT ACA CAC TCA ACC AAG C | H-H7858 | TGC TGT GTA TGC GTC AGG AT |
| L8245 | ACC CAT CAT AGA AGA ACT CC | H7717 | ATG CTT GGT TGA GTG TGT AC |
| L7943 | TCA AAA CGA GAA GTA TCT GCA | IS-H8284 | GGT GTG ATC GTG AAA GTG TAG G |
| L8586 | CTT AAA CCT GGA GAA ATA CGA | H8723 | TTT AGT CGT CCT GGG ATA GC |
| IS-L8189 | GCC CTA TAT GCC TCT ATG GC | I-H8738 | GTC GTC CTG GGA TAG CAT CTG |
| L8597 | AGA AAT ACG ACT ACT AGA AG | 6-H9343 | GAG ATA TAA GTA TTA GTG AT |
| J-L8591 | CCT GGA GAA ATA CGA CTA CT | JS-H9222 | GGG TGT TGG GAA TAG TAA GC |
| 7-L8957 | GGT ATG CCA CAA CTA GAT ACA | H9564 | TAC TGG TTG AAT AAA TAG GC |
| JS-L9052 | CTC ATA CCC AGC AAG CCC AG | J-H9621 | CCC TGC TGT AAT GTT GGC TG |
| K-L9451 | GCA ACC GTA TTC ACA GGA TTC C | KS-H10014 | TGT GGT GGC CTT GGA AAG TGC |
| L9784 | TAC ACG ACA ATA CAT AAT GA | K-H10502 | GCC GAA GTG GTG GTT GGA TG |
| L9831 | AGT AAA CCC AAG CCC ATG AC | 7-H10523 | GTA TCA GGC TGC GGC TTC AAA |
| KS-L9870 | GCC CTT TTA ATA ACA TCA GG | L9129 | AAA ATC TAT TTG CCT CTT TC |
| 8-L10339 | AAT ATT ACG AAG CAC CAT TCA | LS-H10961 | GCT AGG CTT GCT GCT AGT AGG |
| L-L10346 | CGA AGC ACC ATT CAC AAT CTC C | H 10715 | GCG ATT AGT ACG AGT AGG GAG |
| L10614 | CTT CCA ATC AAT CAG TTT CG | H10747 | CAT ATG GGC TTG TTT TTT CT |
| LS-L10869 | CCT TCC CCT ACC ATG AGC ATC C | L-H11398 | GGG TAG TAG TAT TGT TGT TG |
| M-L11230 | CCC TAG CTA ACA TAA TAC CC | MS-H11784 | CGT TCT GTT TGG TTT CCT CAG C |
| L11300 | TAC TAG TAA TAG TAT CCA AC | 8-H11892 | ATT AAG AAG TTT AGT GAG CCT |
| L11435 | AAT GCA ACA GTA CAT AGT CTC C | L11438 | GCA ACA GTA CAT AGT CTC CTC |
| L11627 | AAC CCG AAA AAA ACT AT | MS-L11683 | AAC CTT CAC CGC CAC CGA AC |
| 9-L11720 | CTA TTC GAA GCA ACA CT A GTA | M-H12219 | GCG ATT AAG GAT TTT AGG TCG G |
| N-L12073 | CGG AGG CTA TGG CAT AAT GC | NS-H12623 | GGT GGG TGT ATT TTC CTC GT |
| L11946 | ATT AGC ATG CAT CAT AGC | L12169 | ATA ACC AGC TCT ATC TGC TTA |
| L12254 | TAG CAA TCA TAA TTC AAA CC | H13500 | AAT CAT GCT ATG GAT AGG |
| L12269 | AAG CCT CAC AAA CCT AGC C | H13247 | TGG AGA ATT CTA TAA TTG |
| NS-L12507 | CAG CAT CAT TTT CAT GAT CC | H12293 | TCC TAT GAA ACT TCA GGG |
| L12692 | TAC TAC TGA CCT TAA ACC | N-H13085 | GGG GAC TAG GCT GAG AGT GAA GG |
| L12789 | TTA AAT ATT CTT ATC TAC C | L12996 | AAT ATC CAA CTC AAA CAT C |
| O-L12941 | CCC ATT CGC CTC ACT CAC AT | OS-H13470 | CTC CGA TGC GGT TGT ATA GG |
| 10-L13080 | GTC CCC TTA CTA ATA TTT AT | 9-H13268 | GTC TGA GTG TAT GTA TCA TAT |
| OS-L13333 | CCG CCA ACA ACC TCT TCC AA | O-H13903 | TGG GGT TGG TTG ATG CCG AT |
| P-L13771 | GCC TAG GAG CTA TCA CCA CCT T | PS-H14358 | TTG GTG GGA TGT TGT TGG AG |
| L14005 | CGA AAA ATA GGC GGA CTG T | 10-H14580 | CGG ATT TTT GGC TTA TAG ATA |
| L14162 | TAC TAA TAA CAT TAA TTG CC | 11-L14395 | AAA TAA CAG CCC TAA TCG TAA |
| PS-L14240 | ACG TTT CCC ACC CCT AGT CC | P-H14796 | GGT TGA TCA TTG CTT ATT GG |
| Q-L14522 | CCA AAA ACA ACC TCT TTC ATC C | QS-H15174 | GGG TTG ATT GTA AGT GGT GG |
| L14833 | AGC AGC AAT CCC CAT AGC TTC | Q-H15583 | AGC GAA TAA CTC ATC CGT AA |
| L15007 | TCT TAT TAG AAA CTC AAA CC | H15354 | TTA GTG GGT GTG ATT TTC GG |
| QS-L15084 | CCA AAT AAA TCA AAA ACA CC | H15829 | AAA AGC CCC CTC AGA TTC |
| L15280 | GGA ATC TAA CCA CGA CCA A | RS-H15971 | CCG GTA GGG TTG TTG GAT CCG |
| R-L15439 | TTC GGT TCC CTC TTA GGC AT | H16123 | GGG TTT GCT GGG GTG TAG TTG TCT |
| L15562 | ATT ACG GAT GAG TTA TTC GCT ACC | 11-H15907 | TGA TGA ATG GCA GGA TAA AGT |
| S-L16254 | GCC CAT ACT ACA CAC ATC CA | 12-L15762 | CTG AGG AGC TAC GGT CAT CA |
| RS-L15801 | CCC TTA TAT CGG AAC AGA CC | R-H16379 | GGG TGT TCT ACG GGT TGT CC |
| L16116 | AGC AAA CCC ACT AAA CAC CCC AC | L15971 | AAT CTC ATC AGA CAT AGA C |
| L16575 | AGA CTA ACT CCG CCA TCA GC | H16543 | AGT CTC CTT CCT TGA GTC TTG G |
